# MOBILITY EDUCATION FOR THE DIVERSE AGING COMMUNITY, CAREGIVERS, AND THEIR HEALTH CARE PROVIDERS

**DOI:** 10.1093/geroni/igz038.1089

**Published:** 2019-11-08

**Authors:** Chrysanne Karnick, Ruth Manna, Natalie Gangai, Rosario Costas Muniz, Beatriz Korc-Grodzicki

**Affiliations:** 1 Memorial Sloan Kettering Cancer Center, New York, New York, United States; 2 Memorial Sloan Kettering Cancer Center, New York, United States

## Abstract

Older adults can reduce fall risk in their homes and the community. Health care professionals (HCP) have a role in preventing falls. An interprofessional team of HCPs at a comprehensive cancer center created and delivered educational workshops to increase knowledge about falls prevention. Educational workshops were provided in community centers, libraries, places of worship and at local hospitals to medically underserved, diverse community members, caregivers and HCP. An Occupational (OT) and Physical Therapist (PT) taught three workshops together and the OT taught nine workshops. Workshops included fall prevention, home modifications, safe patient handling (SPH), and the role of OT/ PT in geriatric oncology care. Practical and culturally competent steps were emphasized, with translation of written materials and live interpretation provided as appropriate. Knowledge increase was assessed, and post-session qualitative data was collected. The mean age of community members was 68 years, of nurses was 42, and of caregivers 63. A majority of participants were female. 220 older adults completed surveys, 40 caregivers, and 11 registered nurses. The Falls Prevention workshops with unmatched (n=79) and matched data (n=140) showed significant improvements in knowledge [t(135)=-3.33, p<0.001; t(139)=-4.03, p<.001; respectively). Caregivers who participated in the SPH workshop improved their learning for the unmatched (n=12) and matched data (n=28) after participating in the workshops [t(22)=-3.50, p=.002; t(27)=-3.95, p<.001] respectively. For nurses, the change in scores from pre (M=.56) to post scores (M=.73) were significant (t=-2.76, df=10, p=.02). Caregivers and HCPs benefit from continued education to promote safer, holistic care for family members and patients.

